# Digital health technologies for improving the management of people with chronic obstructive pulmonary disease

**DOI:** 10.3389/fdgth.2025.1640585

**Published:** 2025-08-06

**Authors:** Hye Yun Park, Sunga Kong, Mangyeong Lee, Hyein Ryu, Yoko Hamakawa, Fabrizio Luppi, Janice M. Leung

**Affiliations:** ^1^ Division of Pulmonary and Critical Care Medicine, Department of Medicine, Samsung Medical Center, Sungkyunkwan University School of Medicine, Seoul, Republic of Korea; ^2^ Department of Clinical Research Design and Evaluation, SAIHST, Sungkyunkwan University, Seoul, Republic of Korea; ^3^ Department of Digital Health, SAIHST, Sungkyunkwan University, Seoul, Republic of Korea; ^4^ Center for Clinical Epidemiology, Samsung Medical Center, Sungkyunkwan University School of Medicine, Seoul, Republic of Korea; ^5^ Centre for Heart Lung Innovation, St. Paul’s Hospital, University of British Columbia, Vancouver, BC, Canada; ^6^ Department of Respiratory Medicine, Kyoto University, Graduate School of Medicine, Kyoto, Japan; ^7^ Respiratory Unit, Fondazione IRCCS San Gerardo dei Tintori, Monza, Italy; ^8^ School of Medicine and Surgery, University of Milano-Bicocca, Monza, Italy; ^9^ Division of Respiratory Medicine, Department of Medicine, University of British Columbia, Vancouver, BC, Canada

**Keywords:** COPD, telehealth, digital health, apps, wearable devices

## Abstract

Advances made in digital health in recent years have the potential to improve the care of patients living with chronic obstructive pulmonary disease (COPD) for whom substantial disability still exists. In particular, telehealth and telerehabilitation programs, wearable devices, and apps have been studied as novel methods of providing care to COPD patients who may have limited access to clinical centers or who may benefit from an increased level of monitoring. Many of these interventions gained traction during the COVID-19 pandemic when mandated social isolation required the rapid implementation of remote care models. While these digital health interventions have since demonstrated promise in delivering care to otherwise isolated communities, the ongoing need for more evidence proving their positive impact on important clinical outcomes remains a barrier to their full implementation. How to best integrate digital health solutions into existing care models requires greater consideration of the technological, financial, and labor demands such solutions may entail.

## Introduction

Chronic obstructive pulmonary disease (COPD) is defined as a heterogeneous lung condition due to abnormalities of the airways (bronchitis) and/or alveoli (emphysema). Persistent and often progressive airflow obstruction that marks the disease results in chronic respiratory symptoms (dyspnoea, cough, sputum production, and/or exacerbations) (1). COPD is the third leading cause of death worldwide (2) and it is estimated that there are 480 million people suffering from COPD all over the world (3). By 2050, this estimate is expected to reach 600 million people (3). Compared to the unaffected population, patients with COPD are at higher risk for the development of coexisting extrapulmonary co-morbidities that are associated with poor outcomes, including cardiovascular disease, lung cancer, diabetes, osteoporosis and death (4). Therefore, COPD is associated with a range of potential impairments, including respiratory, physical, social, and emotional, reducing quality of life and limiting daily activities. However, COPD management is complex and an individualized approach to care depends on the condition's severity, including the degree of symptoms, exacerbation frequency and severity, and hospital admissions (1). Targeted COPD medications such as inhalers, while the backbone of therapy, are plagued by slow development (5), thus novel strategies to improve care are desperately needed.

The digital health revolution that has evolved over the past few decades may provide opportunities to improve COPD care. Digital health includes various solutions that utilize digital technologies to meet health needs, including health information technology, telehealth and telemedicine, mobile health, wearable devices, artiﬁcial intelligence, machine learning, the Internet of Things, and digital therapeutics. These technologies have the potential to change how diseases are screened, diagnosed, and followed, allowing for more precise proﬁling of disease progression and thus improving management. This review provides an overview of the best studied digital health opportunities, including its various forms and applications in COPD management. To provide context for emerging digital health technologies in this field, we first offer readers an overview of COPD, from its pathological hallmarks, clinical presentation, and current management strategies both for long-term maintenance care and acute exacerbations. We then narrate the evolution of and evidence for telehealth, telerehabilitation, wearable devices, and apps that have been the most rigorously studied to date in COPD. Finally, we address the substantial barriers that exist that must be addressed prior to the full implementation of digital health technologies in COPD, barriers present at patient, health care provider, and systemic levels. Our narrative review is thus designed to provide both a broad overview of COPD digital health technologies and guidance for future research to improve their implementation in patient care.

## Chronic COPD management

The physiological hallmark of COPD is airway obstruction, which is predominantly the consequence of inflammation and fibrosis of the small airways (chronic bronchitis) and the loss of alveolar attachments (emphysema) resulting in a reduction in forced expiratory volume in 1 s (FEV_1_) and the FEV_1_/forced vital capacity (FVC) ratio. For almost 50 years, COPD has been widely accepted as a self-inflicted condition caused by tobacco smoking (6). COPD was considered a disease occurring in susceptible individuals in whom smoking induced an abnormal inflammatory response (7) that damaged the airways and alveoli, accelerating the physiologic decline of lung function with age. The resulting airflow limitation and chronic respiratory symptoms are difficult to reverse and may periodically be manifested as exacerbations (8). However, recent data suggest that about a third of COPD patients worldwide are non-smokers (9). In addition to genetic disorders such as alpha-1 antitrypsin deficiency, other environmental pollutants, such as smoke from biomass fuel used for cooking and heating (9) and air pollution (10), are also considered major environmental risk factors for COPD in many places around the globe. Moreover, extrapulmonary comorbid conditions are highly prevalent among patients with COPD but are largely unrelated to lung function (4). Therefore, COPD can be seen as the pulmonary component of a systemic and multimorbid syndrome (11).

The Global Initiative for Chronic Obstructive Lung Disease suggests an initial approach to disease management based on the intensity of symptoms and the history of exacerbations, with a subsequent algorithm that includes using blood eosinophil counts for adjustment of therapy (1). A long-acting muscarinic antagonist (LAMA) is the initial drug of choice for patients with mild disease and no exacerbations (1). If the patient has more severe dyspnea, severe airflow obstruction, and lung hyperinflation, combining a LAMA with a selective long-acting beta2-agonist (LABA) is more effective; the two agents can be provided in a single inhaler to simplify treatment (1). It is reasonable to start therapy with a combination of a LABA and an inhaled glucocorticoid in patients with a history of asthma, wheezing, rhinitis, polyps, or allergies; a history of exacerbations; an elevated blood eosinophil count (>150 per cubic millimeter); or a combination of these findings. If exacerbations continue (two or more or one requiring hospitalization), the combination of a LAMA, a LABA, and an inhaled glucocorticoid in a single canister decreases the risk of exacerbations, improves lung function, and may decrease the risk of death (1). Adjunct therapies that have been found to be beneficial in patients with COPD include pulmonary rehabilitation, a multidisciplinary exercise training program that can help to relieve symptoms and improve exercise capacity (12).

## Acute COPD exacerbations

An exacerbation of COPD (AE-COPD) is defined as an event characterized by increased dyspnea and/or cough and sputum that worsens in <14 days which may be accompanied by tachypnea and/or tachycardia and is often associated with local and systemic inflammation caused by infection, pollution, or other insult of the airways (13). AE-COPD are clinically relevant events in the natural history of COPD because they negatively impact health status, lung function, rates of hospitalizations, and disease progression (14). Nevertheless, patients with COPD are also at increased risk of other acute events, particularly decompensated heart failure (15, 16), pneumonia (17), and pulmonary embolism (18, 19) that may also mimic or aggravate an AE-COPD.

Exacerbations are considered to be mild when only worsening symptoms are reported; moderate when the patient receives antibiotics, systemic glucocorticoids, or both; and severe when the patient visits an emergency department or is hospitalized (20). They may also increase the risk of myocardial infarction, stroke, pulmonary embolism, and death (18, 21, 22). A subset of patients with COPD are prone to developing frequent exacerbations (23). Frequent exacerbations are associated with worsening health status, more rapid decline in lung function and are a driver of health care costs, accounting for more than 20% of all readmissions occurring within 30 days after a hospitalization for the same diagnosis (24, 25). Therapies for AE-COPD include short-acting inhaled beta2-agonists and short-acting anticholinergic agents, while systemic glucocorticoids help to improve airflow, gas exchange, and symptoms (26, 27). Antibiotics, particularly for patients with increased sputum purulence and volume, may also reduce treatment failure and improve short-term mortality rates (28). Long-term therapies that can help prevent AE-COPD include chronic macrolides (29) and phosphodiesterase-4 inhibitors such as roflumilast (30).

Despite these drug interventions, many patients with COPD continue to face poor quality of life (31) and reduced lifespans (32). Ultimately, clinicians and allied health providers must navigate the complex journey of both acute and chronic management in COPD within the limitations of these treatments. In the next section, we present the evidence surrounding the most widely studied digital health interventions that have the potential to improve COPD care.

## Telehealth and telerehabilitation

The telecommunications revolution of the 20th and 21st centuries has propelled the telehealth movement forward with extraordinary rapidity. Where once a simple telephone may have been considered the pinnacle technology connecting patient with doctor (33), we now have at our fingertips a plethora of video communication interfaces, electronic medical record systems, and real-time monitoring devices that can instantly transmit important health information to and from a patient. In their ideal forms, these technologies bridge significant divides that may hamper patient care, including physical distance from providers and healthcare facilities and lengthy wait times.

For patients with COPD, who are often elderly, may have considerable respiratory symptoms that limit their ability to attend in-person appointments, and who require complex multidisciplinary management, telehealth may serve a particularly useful role in their care. The introduction of telehealth into COPD management largely occurred around the turn of the century following the expansion of Internet access across the globe and the proliferation of mobile devices. One of the first studies ever to evaluate the potential of telehealth in COPD was conducted in the United Kingdom in the late 1990s where video phones installed in patient homes allowed a nursing team to monitor vital signs, peak expiratory flow rates, and respiratory symptom scores during acute exacerbations (34). While this low-bandwidth technology often did not have sufficient image resolution for the nursing staff to accurately assess respiratory rate, it nonetheless demonstrated the feasibility of a telehealth approach to home monitoring. Subsequently, numerous trials emerged that studied remote monitoring of vital signs in patients with COPD (35) and integrating such monitoring systems with either telephone or video linkages to provider teams (36–39). Although initial assessments of the efficacy of these small programs were promising, with reductions of hospitalizations, emergency room visits, readmissions, and overall health care utilization and costs reported (40–44), the integration of modern technology into COPD care was certainly not universally welcomed. Practitioners in this early phase voiced concerns about the impact technology had on the efficiency of care and on the patient interaction experience, particularly when technical problems disrupted visits (45–48). Studies demonstrating no improvement in the quality of life (49, 50) of patients with COPD also called into question the benefit of these programs.

Proposed models of telehealth in COPD care have included not just these remote monitoring systems connected to a health care team, but also electronic tools to predict the onset of acute exacerbations (51–59), virtual education programs to assist patients with important elements of disease management such as inhaler technique (60–64), and telerehabilitation (65–70). Patient perceptions of these programs has generally rated high, noting convenience (71, 72), self-empowerment (72–74), and sense of security (73–77) as being positive attributes of telehealth. Nonetheless, the growing body of literature on telehealth in COPD has still not yet shown clear benefits when it comes to key outcomes such as exacerbations, hospitalizations, and mortality. Supplementary Table S1 provides an overview summary of relevant trials. A 2013 randomized controlled trial from Scotland (*n* = 128 in the telemonitoring group and *n* = 128 in the usual care group) demonstrated no significant difference in the mean number of COPD admissions over one year nor in the number of days to hospital admission (78). In fact, a subsequent randomized controlled trial published in 2016 evaluating telemonitoring with a link to a hospital-based care team suggested potential harm associated with telehealth: hospital admissions at six months was significantly higher in the telemonitoring group compared to the control group (*p* = 0.026) (79). The PROMETE II trial, a randomized controlled trial comparing home telemonitoring in 237 patients with severe-very severe COPD to routine clinical practice failed to show any benefit in exacerbation rates, hospitalization days, intensive care unit admissions, and quality of life scores (80). Results were similar in the 2018 CHROMED trial which did not find any benefit to telemonitoring in terms of time to hospitalization and rate of exacerbations in 312 patients with COPD (although a significant difference was demonstrated in hospital readmission rate, *p* = 0.017) (81). Multiple studies evaluating cost-effectiveness of such programs also appear to have mixed results, with some studies demonstrating no difference (82–85), improved cost-effectiveness (86–88), and worse cost-effectiveness (89) compared to usual care. Altogether, the evidence compiled would not suggest that telehealth programs provide tangible long-term advantages yet, with the caveat that our understanding of which subgroups of COPD patients may in fact benefit is still quite limited (90).

Of all the methods in which telehealth can be implemented in COPD care, telerehabilitation is perhaps the best studied. Pulmonary rehabilitation in COPD has well-established benefits when it comes to improving health-related quality of life and exercise capacity (91) and reducing mortality following exacerbations (92). In reality, though, access to pulmonary rehabilitation for many patients remains elusive, particularly for those living in rural and sparsely populated regions (93, 94), and utilization of pulmonary rehabilitation remains disturbingly low (95). Delivery of equivalent services through a virtual interface could potentially bring this valuable resource to a greater number of eligible COPD patients. Early evidence of the benefit of such programs was demonstrated in a 2017 multicentre randomized controlled trial evaluating a smartphone-based telecoaching program in 343 patients with COPD (96). Patients in the intervention arm had a greater increase in steps per day and 6-minute walk distance compared to the usual care group. Other studies have since demonstrated that enrollment in a telerehabilitation program can reduce hospital readmissions (67, 70). Subsequent trials comparing home-based to hospital-based rehabilitation programs have demonstrated non-inferiority in terms of reducing the risk of COPD exacerbations and hospitalizations (69) and improvements in 6-minute walk distance (66, 68, 97) and COPD Assessment Test scores (97). To date, no safety issues have been identified with these home-based telerehabilitation programs (98). However, the lack of cost-effectiveness analyses and the wide variability in programs studied are ongoing limitations that should be addressed (98, 99).

Regardless of these inherent gaps in knowledge, the rapid implementation of telehealth in COPD care was necessitated by the COVID-19 pandemic during which in-person assessments, diagnostic testing, and pulmonary rehabilitation programs were drastically reduced or halted entirely overnight. Health care systems were forced to immediately adapt to pandemic conditions in the interests of protecting patients from contracting the virus, with virtual video and telephone consultations substantially increasing in the first year of the pandemic (100, 101). While a full evaluation of these measures is still ongoing, preliminary reports attest to the feasibility of their emergency implementation and the positive reception of such measures in both patients and providers. The majority of clinicians have reported that they were still able to assess symptom severity and provide smoking cessation counseling despite remote care (102), while patients felt positively that telemedicine allowed them to continue accessing health care without being exposed to COVID-19 (103) and provided them with a sense of comfort (104). Still, access to telehealth was patchy in the first days of the pandemic, with one study reporting that only 12.6% of patients with COPD had regular access to medical visits during the lockdown period despite 59.1% of physicians transitioning to telehealth platforms (104). As the experience with virtual platforms grows in health care systems around the globe, telehealth will nonetheless form the foundation of care delivery that will allow for flexibility with fewer disruptions to patient care in times of emergency.

## Wearable devices

The symptoms and conditions of COPD patients including vital signs, oxygen saturation, sleep pattern, and physical activity can be monitored by wearable devices. Unsurprisingly, there has been a sustained demand for the development of tools for symptom monitoring and health management in COPD.

Among wearable devices (Table 1), wristbands and smartwatches are the most commonly used, which can measure blood pressure, heart rate and variability, respiratory rate and variability, oxygen saturation, physical activity, body temperature, metabolic function, sleep indicators, and autonomic nervous system function. Wearables worn on the upper body and chest can also accurately measure heart rate, respiratory rate, and activity level. In particular, chest-worn wearables have the advantage of being able to collect more accurate data during exercise or daily activities. Wearables worn on the finger can measure sleep patterns, heart rate, and body temperature, and are useful for monitoring the quality of sleep and overall health status of the user. Each type of wearable device plays an important role in monitoring and managing COPD patients. Wearables for monitoring vital signs track basic indicators of health status in real time, and physical activity wearables help enhance the user's activity level and facilitate various exercise programs.

**Table 1:** Types of wearable devices.

Type	Measurement data	Wearable device examples
Wristbands, Smartwatches	Blood pressure	Charge HR (105, 106)
	Heart rate and variability	Charge 2 (106, 107)
	Respiratory rate and variability	Omron Walking Style3 (108)
	Oxygen saturation	Garmin Vivofit2 (108, 109)
	Activity level	Apple Watch 6 (110)
	Body temperature	Apple Watch 7 (108)
	Metabolic function	
	Sleep indicators (Sleep Stages, Sleep Duration, Sleep Score, Awake Times, Sleep Efficiency)	
	Autonomic nervous system function	
Rings	Sleep pattern	OURA Ring (111)
	Heart rate	RingConn Smart Ring
	Body temperature	Ring AIR
	Oxygen saturation	
	Physical activity	
Vests, Shirts, Upper body bands, Waist bands	Electrocardiogram, heart rate, heart	Fitbit One (109)
	rate variability	Hexoskin (109)
	Respiratory rate	Master Caution
	Physical activity	
Accelerometers and Chest bands	Respiratory rate	Zephyr BioHarness (112)
	Electrocardiogram and heart rate	Garmin HRM-Pro Plus
	Physical activity	

Research prior to 2019 primarily focused on the validity and accuracy of sensor-based remote monitoring devices compared with gold standard medical equipment (113, 114). Subsequent studies aimed at using these devices to develop a prediction model of COPD exacerbations by collecting short-term (30 days) (115) and long-term (90 days) patient monitoring data (116). The feasibility of continuously and reliably capturing audio, heart rate, and physical activity through smartwatches (117), moreover using these devices to provide education and self-monitoring interventions (118), were also demonstrated in COPD patients. Rubio et al., for example, showed the potential of using home monitoring of resting breathing rate data to supervise recovery from COPD exacerbations (116). Monitors were able to provide accurate assessments of breathing rate, although some patient feedback criticized the monitors for being intrusive during the acute illness phase.

Although promising, the widespread adoption of wearable devices by patients with COPD has not been universal, hampered by a lack of evidence that these devices can meaningfully improve important clinical outcomes, inaccessibility, and inaccuracy concerns. Walker et al., reported that remote monitoring of lung function by forced oscillation technique and cardiac parameters did not change time to first hospitalization and quality of life over 9 months in a randomized controlled trial (81). A survey for devices listed up to June 2019 reported that the devices with the most technological promise and compatibility with daily living had high or unlisted prices, placing them out of reach for many patients (119). While studies by Buekers et al. (114) and Chan et al. (120) monitored oxygen saturation using consumer-based wearable finger and smartphone oximeters, technical limitations were reported, including incomplete data recording during moderate to vigorous physical activity and exercise. As noted in a systematic review, which compiled data from seven papers published prior to 2021, oxygen saturation and respiratory rate devices were valid compared to other respiratory monitoring devices, but were not accurate in predicting exacerbation events (121). Meanwhile, although Pipek et al. demonstrated that a wearable device (Apple Watch 6) can be a reliable way to obtain heart rate and oxygen saturation in patients with lung diseases in a controlled environment (110), subsequent publications called into question these findings. A study conducted by Stove et al. raised concerns that wearable devices such as the Apple Watch Series 7 and Garmin Vivosmart 4 should not be relied upon for monitoring oxygen saturation during pulmonary rehabilitation. Specifically, their research revealed that these devices tended to overestimate oxygen saturation levels in individuals with COPD when levels were below 95%, while underestimating oxygen saturation when levels were above 95% at rest and immediately after the 30 s sit-to-stand test and the 6 min walk test (108).

Other research followed utilizing consumer-based activity trackers as tools for measuring physical activity (113) and as coaching devices for promoting physical activity (122) in patients with COPD. The initial studies focused on evaluating these devices' accuracy and feasibility. In a single-arm feasibility study, 19 patients with COPD wore a commercial activity monitor every day (median 42 days) during pulmonary rehabilitation. These patients were able to increase their exercise steps throughout the pulmonary rehabilitation process without sacrificing non-exercise physical activity. These patients demonstrated improved dyspnea and quality of life following use of the device, confirming that wearable technology can support effective remote walking exercise prescriptions and engagement during pulmonary rehabilitation (123). However, in another study involving 122 patients divided into standard care, self-monitoring, and telemonitoring groups, COPD patients in the remote or self-monitoring groups did not experience improved outcomes or reduced healthcare utilization compared to standard care, despite regular use of technology. This was attributed to low primary healthcare utilization, the absence of structured training components, and inadequate integration of technology with action plans (124).

More recently, attempts to integrate physiological features captured by wearable devices with environmental monitoring to read toxic and noxious exposures have aimed to improve our abilities to predict COPD exacerbations. For instance, Wu et al., used a wearable device, home air quality sensing device, a smartphone app, and a supervised prediction algorithm to achieve excellent performance (accuracy of 92.1%) in predicting whether a patient would experience an acute exacerbation of COPD within the next seven days (125). These more sophisticated algorithms integrating multiple technologies may prove beneficial in future, yet at present clinicians remain somewhat mixed on their use in clinical practice. In one mixed-method survey, most clinicians attested to not employing these technologies routinely in their patient panels and reported low confidence in their overall effectiveness (109). Healthcare practitioners report a lack of guidance as to how to implement these technologies in their clinical practices and seek more information on their use (105). As one scoping review has outlined, strategies in addition to technology are needed to effectively support health management and physical activity monitoring in COPD patients (126). Specifically the authors note these key areas that must be addressed and achieved by wearable technology innovators: (1) Monitoring and tracking activity and health, (2) Enhancing motivation for physical activity, (3) Ensuring acceptability of the device, (4) Addressing technical issues with the device, (5) Establishing appropriate and achievable health goals, (6) Integrating the device into daily life and routine, and (7) Recognizing the physical and psychological benefits of device usage (126). Physiological data measurement during exercise remains inaccurate with evidence coming from small trials, and current health behavior theories are not yet well integrated into self-management, resulting in low effectiveness. In future, large-scale studies focusing on multiple outcomes will be required, and the release of consumer-based wearable devices equipped with predictive models remain goals for the community.

## Apps

Today, mobile health (mHealth) apps, either on computer, tablet, or smartphone interfaces, are used for a wide variety of health services and for patient education. In their ideal form, these apps can help patients monitor their COPD, manage their symptoms, identify warning signs that should trigger a higher level of care, and receive education and treatment plans. From 2014 to 2017, research primarily focused on the design and the feasibility of these apps. Programs delivered through apps were provided to patients through a combination of online and offline methods, through both Web and phone interfaces, and designed to promote their physical activity (127–129). These studies demonstrated encouraging results that these interventions could improve physical activity levels in patients with COPD, and that both patients and physiotherapists had positive attitudes towards the apps. Nonetheless, demonstrating concrete improvements in outcomes such as symptom reduction or hospitalizations was elusive. For instance, Farmer et al. performed a randomized controlled trial in 2017 using a tablet-based system that included a symptom diary, vital sign monitor, and education modules, but did not find any benefit in terms of patients' St. George's Respiratory Questionnaire scores or hospital admission rates (130). A subsequent systematic review of apps during this era demonstrated only a weak association with a lower risk for hospital admission and no significant impact on length of hospitalization (131). Other systematic reviews noted that health-related quality of life and physical activity could be improved by smart technology apps, but that these effects were not sustained over longer periods of time (132).

With sensor modules that can detect changes in vital signs now being integrated into smartphone technology, though, the promise of apps to predict adverse outcomes in COPD such as exacerbations may be on the horizon. Apps that allow patients to log their symptoms daily in conjunction with heart rate and oxygen saturation monitoring that could alert a patient to worrying trends may conceivably allow early detection of exacerbations and therefore prompt immediate treatment and prevention of hospitalizations. Early trials suggested that these apps were feasible with excellent compliance rates amongst patients who would be required to report their symptoms on a regular basis (133–135). Interestingly, these apps can capture a far greater number of clinical worsening events than patients ultimately recalled themselves and reported to their doctors (136). Criner et al. demonstrated that one such app could shorten the time to which a patient could receive clinical direction from their primary care provider for an exacerbation event (135). The EmmaCOPD app which monitors both symptom worsening and step count decline to prompt a clinical intervention resulted in a reduction in the number of hospitalized days and exacerbation frequency (137). Further machine learning models utilized in another trial by Chmiel et al. allowed a digital health app to use symptom data to predict an exacerbation event within three days with a sensitivity of 0.551 and a specificity of 0.759 (138). No doubt, these algorithms will need to be refined in future as technologies develop to improve risk prediction models.

More evidence that apps can be successfully integrated into COPD management with beneficial results have come in the domain of pulmonary rehabilitation. The Kaia COPD app containing an exercise training program was recently demonstrated in a randomized controlled trial to significantly improve symptom scores and allowed patients to maintain physical activity after completing pulmonary rehabilitation courses (139). These promising results in improving exercise in patients with COPD compared to control groups have been reflected in other trials as well (140, 141). Mobile apps also appear to be effective in empowering patients through education. Both quantitative assessments and qualitative interviews with patients following an mHealth app intervention, for example, found significant improvements in disease awareness and self-efficacy (142). Coping abilities and knowledge of COPD management have also been reported to increase following mHealth app use (143, 144).

In general, patient attitudes towards the use of apps in their COPD care are positive (145), but in reality they are faced with a number of choices available on public markets which may lack oversight by health care professionals. Thirteen COPD-related apps were reviewed by Quach et al. in 2023, who found that none of the apps reported data that supported their efficacy (146). Nine of the apps were furthermore found to have outdated or incorrect information about COPD. Only three of the apps provided clear descriptions of their data security features. The medical community must therefore be aware of the proliferation of apps that might in fact be harmful for patients and only apps that have been carefully screened, demonstrate evidence of benefit, and guarantee privacy and security should be promoted to patients. Not only should these apps be constructed with input from health professionals who can ascertain the quality of the information provided, but they should also be the product of a close patient-provider-technology partnership to ensure a patient-oriented design. Iterative multidisciplinary processes in app design where patients are consulted along each step have been described, which may provide the best chance at successful acceptance of apps in patients with COPD (147, 148). These partnerships may also be able to address issues such as poor readability and a lack of cultural sensitivity that have been reported with certain apps (149).

**Figure 1:**
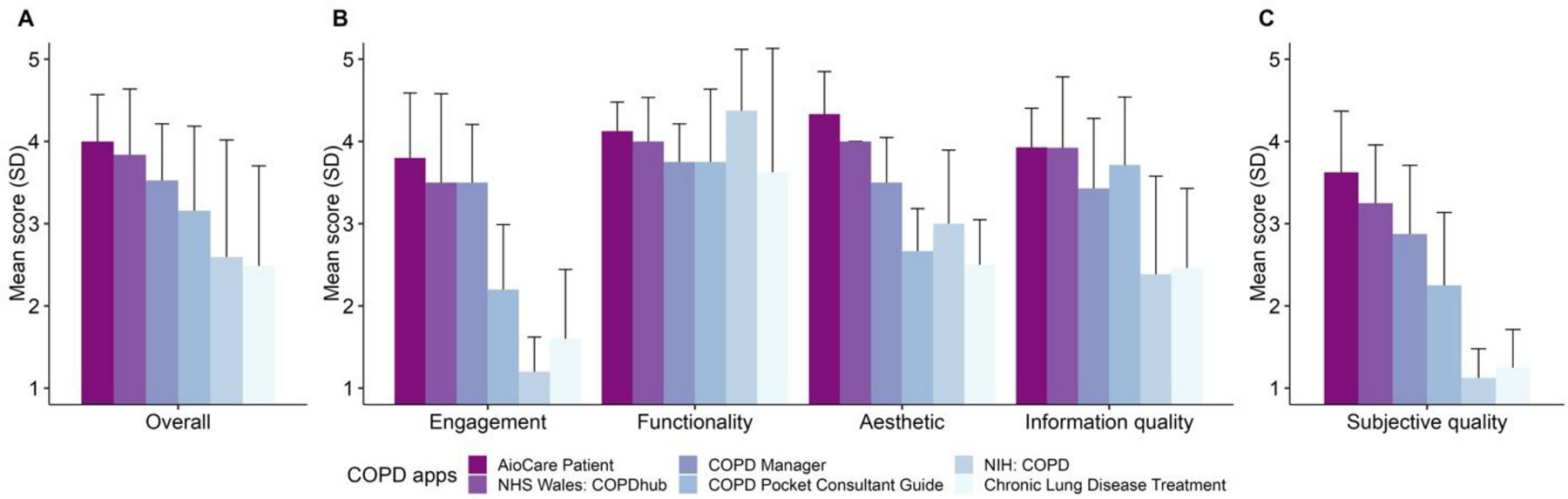
COPD apps by MARS scores. **(A)** Indicates overall app quality by the MARS score. **(B)** Indicates scores by individual objective domains, including engagement (covering fun, interest, and interactivity), functionality (covering performance, usability, and navigation), aesthetics (covering layout, graphics, and visual appeal), and information quality (covering accuracy, credibility, and evidence bias). **(C)** Provides the MARS score related to subjective quality. Error bars indicate standard deviation. All questions were evaluated using a 5-point Likert scale.

The quality of mHealth apps can be assessed using the Mobile App Rating Scale (MARS) which consists of 23 items covering four objective quality dimensions (engagement, functionality, aesthetics, and information quality) and one subjective quality scale (150). With this comprehensive scale, two assessors on our team independently evaluated the quality of six accessible and downloadable COPD apps (AioCare Patient, COPD Manage, NIH: COPD, NHS Wales: COPDhub, COPD Pocket Consultant Guide, and Chronic Lung Disease Treatment). Inter-rater reliability measured by the Intraclass Correlation Coefficient (ICC) was 0.76 (95% CI 0.67–0.83), indicating good reliability. We found that COPD apps tended to focus most on functionalities such as performance, navigation, or usability (Figure 1). However, these apps showed relatively low quality in the engagement dimension such as fun, interest, or interactivity. The focus on clinical information accuracy and self-management functionality in COPD mHealth applications potentially overlooks a critical aspect of user interest. Thus, integration of enticing engagement and gamification elements into app architecture may be necessary for sustaining long-term user adherence and optimization of therapeutic outcomes.

## Barriers and controversies in digital health

**Figure 2:**
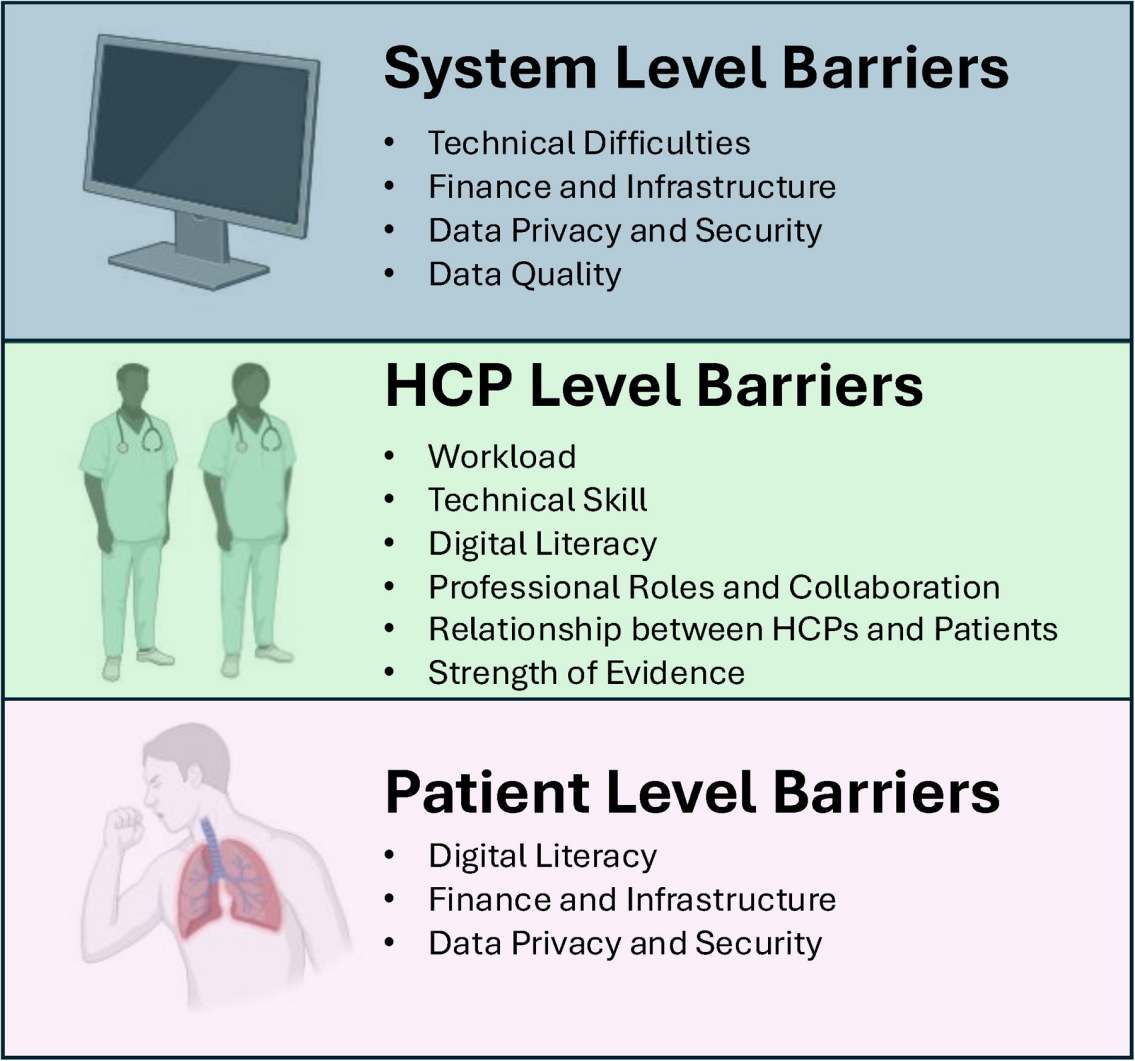
Levels of barriers to digital health. Created using Biorender.

Despite the rapid development and evolution of digital technologies in the health care sphere, the barriers that prevent their full implementation in COPD remain relatively fixed over time (48, 151–157). Until these barriers are effectively addressed by our community, it is likely that digital health will remain at the fringes of COPD care. Here, we consider barriers to digital health arising from three levels of care: those related to systemic and technical problems and those at the level of healthcare providers (HCPs) and patients (Figure 2).

### HCP level barriers

For HCPs, it is essential that digital technologies be seamlessly integrated into what is already a demanding and strenuous workload (151–155). HCPs see themselves as battling a “sea of data,” (155) therefore management of patients' data taken via digital health devices as a part of their daily routine may be perceived as yet another burden to their workload. Devices which constantly monitor patients' oxygen saturation, heart rate, temperature, and symptoms related to COPD exacerbations produce unprecedented amounts of data. A high degree of triggered alarms turn out to be false, resulting in unnecessary clinical work (158). HCPs have voiced concerns about the sustainability of digital health given these added burdens (159). Whether new artificial intelligence technologies can assist HCPs in making sense of these burgeoning datasets is still unclear. Ultimately, appropriate staffing support and compensation would certainly have to be addressed for sustainability to be achieved. Who and what entities will shoulder the financial and technical burden of infrastructure, data management, privacy and security, and quality control has yet to be worked out by the majority of health care systems (151, 153, 154).

Technical skill (151, 153, 154) and digital literacy (151) are also well recognized barriers to full implementation. For many HCPs, the digital frontier represents a shift in practice for which insufficient training has been provided. Facility in using these devices, comfort in addressing technical problems when they arise, not to mention keeping up to date with their latest advances, are all required for HCPs to fully adopt digital health into their practices. At the same time, feedback from tele-rehabilitation programs also suggests that HCPs feel discomfort with the type of care provided through advanced technologies, that particular communication skills are required to master the provider-patient relationship in this novel form (151, 153, 154). When care is provided through screens, HCPs have noted the loss of personal interactions, social contexts, and direct contact through touch and sight, all of which they place value in guiding their understanding of each patient (151, 154). Without this critical accompanying information, concerns regarding safety are additionally raised. HCPs voice worry about providing rehabilitation in remote settings without the guidance of direct observation which may make them blind to each patient's particular safety needs and physically unable to help them if the patient worsens (153). In the face of these limitations, HCPs consider the evidence for digital health technologies in improving COPD outcomes insufficient to counter their apprehensions (155). As more is learned about digital health and better training is provided to HCPs on digital health technologies, fear of and doubt over the unfamiliar may recede. According to Sharma et al. (154), the introduction of telehealth can be experienced as threatening to HCPs, but at the same time HCPs who had experience in providing telehealth had fewer and milder concerns. This suggests that this unfamiliarity to digital health may be overcome through suitable training and support.

### Patient level barriers

In a certain sense, patient concerns with respect to digital health mirror those of their HCPs. Inadequate facility with and access to digital technologies and concerns about data confidentiality and security impact digital health uptake as much for patients as it does for HCPs. First and foremost, uptake requires a certain degree of overall health literacy, defined as “the degree to which individuals have the capacity to obtain, process, and understand basic health information and services needed to make appropriate health decisions” (160). Health literacy, though, remains low in patients with COPD (161–163). This is particularly true for vulnerable groups, such as those of older age, with low education and income levels, and from minority and underrepresented communities (162–165). Those with low health literacy have been shown to be less engaged in self-management plans (163). Even if a minimum level of health literacy is attained, though, further literacy in digital technologies is still needed. Electronic health (eHealth) literacy is defined as “a patient's capacity to seek, locate, understand and evaluate health information from the Internet and apply such knowledge when addressing or solving a disease-related health and concern” (166). Many of the same risk factors (e.g., older age, low income and education levels) for poor health literacy also contribute to poor eHealth literacy (167, 168). Patients with COPD have been shown to have low to moderate eHealth literacy (169, 170), with technophobia, a negative attitude towards aging, lower perceptions of self-efficacy, greater severity of COPD, low technology use, low income, and low education levels significantly associated with worse eHealth literacy scores (169–172). Efforts to increase digital health uptake must therefore address these particular risk factors.

Ultimately, any digital health technology has to be usable for a patient (173). Previous qualitative investigations have noted that patients with COPD often think that telehealth screen fonts are too small for them to read (174, 175) and that touchscreens can be difficult to use particularly if they had manual dexterity problems (176). Excessive keys and buttons can overwhelm users and cause confusion (175). Even the act of downloading an app or creating a login and account can be problematic for users with little experience with smart technologies (173, 177). Technology design that is performed with ongoing, iterative contributions from end-users is thus essential, as is continuous usability testing to ensure that even those with limited eHealth and digital literacy can still navigate them easily. Additionally, to address usability problems, designers must identify where dissonance appears between a system's intended use and the users' actual interpretation and experience in both physical and cognitive aspects. Naranjo-Rojas et al. propose “think-aloud” methods that allow participants to vocalize their concerns and challenges as they use technologies in real time to facilitate the improvement of digital health technologies (177). Displays and interfaces that are accessible, intuitive, organized, and visually appealing are thus essential elements of digital health design.

Access to necessary technologies also remains patchy amongst patients with COPD, reflecting the inequities that exist in the digital health realm. Melzer et al. reported that only 54% of veterans with COPD had a computer at home, only 54% had a smartphone, and only 36% had reliable in-home Internet, resulting in 25% having overall low use of technology (169). Other groups have reported even lower rates of technology ownership. For instance, Alwashmi et al. reported that only 23% of their cohort of patients with COPD owned a smartphone (173). Compounding these inequities are often financial pressures as medical technologies designed for remote patient monitoring carry either high or undisclosed costs that may be out of range for many patients (178). It is not surprising then that patients have expressed they cannot afford the costs that come with digital health technologies (173). Unless greater availability of emerging technology is granted to a wider swath of the COPD patient population, much of the digital health revolution will bypass them.

### System level barriers

Healthcare systems, from the limitations of their technological capacities to the complexities of their organizational and financial structures, also pose significant barriers to the implementation of digital health. While there are no structured articles that have investigated systemic barriers specifically pertaining to digital health in COPD management, key themes emerge in the current literature that highlight the structural limitations facing the digital health revolution. First, we must ask whether our current technologies are sophisticated enough to provide smooth and seamless services to patients living with COPD. Second, are the vast number of health care organizations, all of whom may have differing attitudes towards digital health, budgetary constraints, and their own unique electronic medical systems, ready to implement new digital health programs and technologies? Finally, can healthcare systems ensure the proper data security and privacy provisions that patients will rightfully demand as these technologies are implemented? These concerns are not unique to COPD care, but reflect larger barriers that must be overcome for the entire field of digital health.

Internet accessibility and stability are certainly barriers in promoting digital health. Smaradottir et al. pointed out in an evaluation of a tablet-based telemedicine program for COPD that lapsed data transmission and poor quality image and sound during videoconferencing, due to unstable and insufficient mobile network coverage, were significant system level problems impacting the use of the program (179). Other qualitative studies have echoed these technical concerns, with patients voicing that malfunctioning technology increased their stress and was a barrier to adherence (180). These are concerns not just of patients, but also of HCPs (181). The stability and accessibility of internet service are especially critical when HCPs monitor vital signs in remote settings. Even if the internet infrastructure itself functions properly, digital health technologies cannot work efficiently if programs are incompatible with older operating systems that some patients may have (152).

As we have previously mentioned, digital health solutions must be integrated into existing and already high workloads for physicians and allied health care professionals. However, equally important is integration into existing practice cultures, where the adoption of digital health programs may not be welcome by all and where previously defined clinical roles may now be thrown into question by new technologies. Interviews with focus groups have revealed that the introduction of telehealth systems can result in perceptions that previously valued skills and expertise were now being undermined and replaced (154). Others have noted conflicts between those championing digital health technologies and hospital management and other HCPs who may be more resistant (153). Compounding these issues is the need to adapt existing technologies to a wide plethora of discrepant electronic health record systems, with each healthcare system or hospital having their own unique platforms (182).

Resolution of these conflicts will be necessary before full implementation of digital health in COPD is achieved. Moreover, prioritizing newer technologies obviously demands significant financial resources, which many healthcare systems may be too strapped at present to promise investment. Who would bear these financial responsibilities and how digital health services will be integrated into current medical billing systems to ensure fair compensation remains in question. Conflicting evidence in the literature that providing telehealthcare to patients with COPD may or may not in fact be very cost-effective is yet one more argument against the implementation of digital health solutions (183, 184). A recent 2024 systematic review of randomized controlled trials (184) found that in studies that conducted full economic analyses, the incremental cost-effectiveness ratio of interventions such as electronic patient diaries, real-time monitoring, and teleconsultations was between 3530.93€ to 286,369.28€/quality-adjusted life year. Real-time monitoring and teleconsultations appeared to be the most cost-effective interventions, while electronic patient diaries had a less consistent signal for cost-effectiveness. Nonetheless, study comparisons were hampered by the diversity in methods for cost evaluation and time period of assessment, as well as by many studies having only performed partial economic analyses that solely took into account the cost of the intervention rather than the consequences of the intervention. The authors also noted that most studies have been performed in high income countries, limiting generalizability to low and middle-income countries. These limitations demonstrate the need for further work on the cost-effectiveness question.

Maintaining these digital health systems and preventing them from cybersecurity threats (185) is not just an important factor in promoting digital health to HCPs, but also an issue that has been identified by patients as critical to their support of these new solutions (186). Both patients and providers must feel secure that data remain private and confidential in an age where cyberattacks against health care systems have become increasingly common, disruptive, and costly for all. This requires collaborative work between device and app developers, HCPs, and information and technology cybersecurity experts (185), as well as with regulatory bodies. Both the European Union and the U.S. Food and Drug Administration, for instance, have issued guidance on cybersecurity requirements for medical devices (187, 188). Despite these guidelines, though, developers must continually adapt to a rapidly changing security landscape and ensure that their technologies remain ahead of threats. Contingency plans for when data are compromised would certainly help to improve management of these unfortunate breaches. A separate but related issue would be the question of legal liability associated with these digital health solutions. Licensure and jurisdiction for telemedicine, informed patient consent, maintenance of quality control standards and records, data ownership rights, conflicts of interest, and malpractice have all been raised as legal issues that require concrete guidance in order for digital health solutions to be fully implemented (189–193).

## Future directions for digital health in COPD

The barriers facing the digital health revolution are complex and unsolved at this moment and are certainly not unique to COPD. Large-scale interventions to healthcare systems require time, effort, cost, and significant cultural shifts. Marwaha et al. suggest nine key factors that all healthcare systems and providers should comprehensively consider prior to adopting digital health solutions: production selection, financial value, clinical value, data asset requirements, internal champions, executive sponsors, institutional priorities, implementation resources, and long-term operational strategies (194). Using this kind of scheme will help us to select and implement the most effective digital tools into our daily practice. Patients, health care providers, and health care systems will ultimately require far more rigorous research into the efficacy of these technologies. Do they substantially improve outcomes in metrics that are meaningful to patients living with COPD, such as quality of life, symptom burdens, hospitalizations, and mortality? Will they significantly reduce the enormous cost burden of COPD care that many health jurisdictions now face or will be facing in the coming decades? Answering these questions will certainly require more randomized controlled trials powered to clinically important outcomes with subsequent cost-effectiveness analyses. However, they will also require close partnerships between researchers and end-users, both at the patient and HCP levels. Novel technologies will not be successfully implemented without ongoing iterative input from these stakeholders to ensure they address their needs and technological capabilities.

In due time, we may face yet another pandemic where we will be required to treat patients remotely again. Also, as society ages, there will no doubt be an increasing number of patients who cannot commute to healthcare facilities, necessitating a wider adoption of telehealth solutions. Overcoming these current challenges will lead us a better healthcare system in which providers, patients, and digital health technologies can work together in harmony.
