# Supplementary material for: Digital health technologies for improving the management of people with chronic obstructive pulmonary disease
Source: Front Digit Health. 2025 Aug 6;7:1640585. doi: 10.3389/fdgth.2025.1640585 (PMC12365816; doi:10.3389/fdgth.2025.1640585)
Supplement: Supplementary file 1 [file Table1.pdf]

Digital health technologies for improving the management of people with chronic obstructive pulmonary disease

Supplementary Table

Hye Yun Park,[1] Sunga Kong,[2,3] Mangyeong Lee,[4,5] Hyein Ryu,[4] Yoko Hamakawa,[6,7]
Fabrizio Luppi,[8] Janice M. Leung[6,9]

[1]Division of Pulmonary and Critical Care Medicine, Department of Medicine, Samsung Medical Center, Sungkyunkwan University School of Medicine, Seoul, South Korea.
[2]Patient-Centered Outcomes Research Institute, Samsung Medical Center, Seoul, South Korea.
[3]Department of Clinical Research Design and Evaluation, SAIHST, Sungkyunkwan University, Seoul, South Korea.
[4]Department of Digital Health, SAIHST, Sungkyunkwan University, Seoul, Korea.
[5]Center for Clinical Epidemiology, Samsung Medical Center, Sungkyunkwan University School of Medicine, Seoul, Korea.
[6]Centre for Heart Lung Innovation, St. Paul's Hospital, University of British Columbia, Vancouver, BC, Canada
[7]Department of Respiratory Medicine, Kyoto University, Graduate School of Medicine, Kyoto, Japan
[8]Respiratory Unit, Fondazione IRCCS San Gerardo dei Tintori, Monza, Italy and School of Medicine and Surgery, University of Milano-Bicocca, Monza, Italy
[9]Division of Respiratory Medicine, Department of Medicine, University of British Columbia, Vancouver, BC, Canada

Supplementary Table 1. Telehealth and Telerehabilitation Trials

Author	Year of Publication	N	Trial Design	Intervention	Primary Outcome	Effect Size	Primary Outcome Result		
							Favors Digital Health	No Benefit	Favors Usual Care
Pinnock H (1)	2013	256	RCT	Telemonitoring vs Usual Care	Time to hospital admission for COPD exacerbation after 1 year	Adjusted hazard ratio 0.98 (95% CI 0.66-1.44)			
Chatwin M (2)	2016	68	Randomised crossover trial	Telemonitoring vs Usual Care	Time to hospital admission for acute exacerbation	77 days (telemonitoring) vs 77.5 days (usual care), p=0.189			
Demeyer H (3)	2017	343	RCT	Telecoaching vs Usual Care	Change in physical activity measured by accelerometry	Increase of 1469 steps/day in telecoaching group compared to usual care (p≤0.001)			
Vasilopoulou M (4)	2017	147	RCT	Telerehabilitation vs Usual Care	Rate of moderate-severe acute exacerbations, hospitalizations for acute exacerbations, and emergency room visits	Incident rate ratios: Acute exacerbations: 0.517, 95% CI 0.389–0.687; p<0.001; Hospitalizations: 0.189, 95% CI 0.100–0.358; p<0.001; Emergency room visits: 0.116, 95% CI 0.072–0.185; p<0.001			
Ancochea J (5)	2018	229	RCT	Remote Patient Monitoring vs Usual Care	Proportion of group experiencing a severe exacerbations over 1 year	60% in remote patient monitoring vs 53.5% in usual care, p=0.321			

Walker PP (6)	2018	312	RCT	Telemonitoring vs Usual Care	Time to first hospitalization	224 days (IQR, 209–240 d) in telemonitoring group vs 254 days (IQR, 240–270 d) in the usual care group (p =0.342)			
					EuroQoL EQ-5D score	0.637 in telemonitoring group vs 0.640 in usual care group (p=0.915)			
Bhatt SP (7)	2019	240	RCT	Telerehabilitation vs Usual Care	30-day all-cause readmission rate	6.2% in telerehabilitation group vs 18.1% in usual care group (p<0.013)			
Hansen H (8)	2020	134	RCT	Telerehabilitation vs Conventional Pulmonary Rehabilitation	Change in 6 minute walk distance	Between group difference 8.3m (95% CI -7.7 to 24.3)			
Zanaboni P (9)	2023	120	RCT	Telerehabilitation vs Usual Care	Number of hospitalizations and emergency department presentations	1.18 events per person-year in telerehabilitation group vs 1.88 events per person-year in usual care group (p<0.001)			

Green boxes demonstrate that the digital health intervention improved the primary outcome. Grey boxes indicate that the digital health intervention had no positive or negative effect on the primary outcome. None of the listed trials reported that the digital health intervention was inferior to usual care.

Abbreviations:

RCT: Randomized Controlled Trial

CI: confidence interval

References

1. Pinnock H, Hanley J, McCloughan L, Todd A, Krishan A, Lewis S, et al. Effectiveness of telemonitoring integrated into existing clinical services on hospital admission for exacerbation of chronic obstructive pulmonary disease: researcher blind, multicentre, randomised controlled trial. Bmj. 2013;347:f6070.
2. Chatwin M, Hawkins G, Panicchia L, Woods A, Hanak A, Lucas R, et al. Randomised crossover trial of telemonitoring in chronic respiratory patients (TeleCRAFT trial). Thorax. 2016;71(4):305-11.
3. Demeyer H, Louvaris Z, Frei A, Rabinovich RA, de Jong C, Gimeno-Santos E, et al. Physical activity is increased by a 12-week semiautomated telecoaching programme in patients with COPD: a multicentre randomised controlled trial. Thorax. 2017;72(5):415-23.
4. Vasilopoulou M, Papaioannou AI, Kaltsakas G, Louvaris Z, Chynkiamis N, Spetsioti S, et al. Home-based maintenance tele-rehabilitation reduces the risk for acute exacerbations of COPD, hospitalisations and emergency department visits. Eur Respir J. 2017;49(5).
5. Ancochea J, García-Río F, Vázquez-Espinosa E, Hernando-Sanz A, López-Yepes L, Galera-Martínez R, et al. Efficacy and costs of telehealth for the management of COPD: the PROMETE II trial. Eur Respir J. 2018;51(5).
6. Walker PP, Pompilio PP, Zanaboni P, Bergmo TS, Prikk K, Malinovschi A, et al. Telemonitoring in Chronic Obstructive Pulmonary Disease (CHROMED). A Randomized Clinical Trial. Am J Respir Crit Care Med. 2018;198(5):620-8.
7. Bhatt SP, Patel SB, Anderson EM, Baugh D, Givens T, Schumann C, et al. Video Telehealth Pulmonary Rehabilitation Intervention in Chronic Obstructive Pulmonary Disease Reduces 30-Day Readmissions. Am J Respir Crit Care Med. 2019;200(4):511-3.
8. Hansen H, Bieler T, Beyer N, Kallemose T, Wilcke JT, Østergaard LM, et al. Supervised pulmonary tele-rehabilitation versus pulmonary rehabilitation in severe COPD: a randomised multicentre trial. Thorax. 2020;75(5):413-21.
9. Zanaboni P, Dinesen B, Hoaas H, Wootton R, Burge AT, Philp R, et al. Long-term Telerehabilitation or Unsupervised Training at Home for Patients with Chronic Obstructive Pulmonary Disease: A Randomized Controlled Trial. Am J Respir Crit Care Med. 2023;207(7):865-75.
